# Production of Bio-omega-3 eggs through the supplementation of extruded flaxseed meal in hen diet

**DOI:** 10.1186/s12944-015-0127-x

**Published:** 2015-10-09

**Authors:** Muhammad Imran, Faqir Muhammad Anjum, Muhammad Nadeem, Nazir Ahmad, Muhammad Kamran Khan, Zarina Mushtaq, Shahzad Hussain

**Affiliations:** Institute of Home and Food Sciences, Faculty of Science and Technology, Government College University, Faisalabad, Pakistan; Department of Dairy Technology, University of Veterinary and Animal Sciences, Lahore, Pakistan; Department of Food Science and Nutrition, College of Food and Agricultural Sciences, King Saud University, Riyadh, Saudi Arabia

**Keywords:** Hot extrusion, Linseed, Hen diet, Designer eggs, Omega-3 fatty acids

## Abstract

**Background:**

The full-fat flaxseed meal has obtained relatively new flourished concept as staple chicken feedstuff for the production of designer eggs. However, unprocessed flaxseed also encloses well documented anti-nutritional factors which are associated with growth depression of laying hens. The present research work was carried out to evaluate the impact of full-fat extruded flaxseed meal supplemented diets on productivity performance of hens and production of modified ω-3 fatty acids-enriched eggs.

**Methods:**

The full-fat flaxseed meal was extruded at barrel exit temperature (140 °C), screw speed (160 rpm) and feed rate (25 kg/h) for reduction of anti-nutritional compounds. One hundred and sixty, Babcock hens (age 24 weeks old) were selected at random from a large flock and ten hens were placed in each of 16 wire-mesh pens. The experimental diets prepared by supplementation of extruded flaxseed at 10 %, 20 % and 30 % level were fed to hens along with control.

**Results:**

The extruded flaxseed contained 86 % and 76 % less hydrocyanic compounds and tannin, respectively than the initial material. The hens fed with control diet consumed more feed, possessed heavy body weight and showed higher egg production as compared to hens fed on extruded flaxseed supplemented diets. The loss in body weight and egg production was recorded less for hens fed on 10 % extruded flaxseed supplemented diets as compared to those fed on 30 % extruded flaxseed supplemented diets. None of the experimental diets resulted in significant increase or decrease the total lipids and cholesterol content in egg yolk of hens. The extruded flaxseed supplemented diets resulted in a significant improvement of α-linolenic and docosahexaenoic acid in egg yolk with a concomitant reduction in arachidonic acid. The sensory scores were assigned higher to control eggs. Increasing level of extruded flaxseed in experimental diets decreased the scores for all sensory attributes of eggs.

**Conclusion:**

The present study suggested that extruded flaxseed meal up to 20 % can be supplemented in the diets of hens for maximum deposition of α-linolenic acid and docosahexaenoic acid in egg yolk with acceptable detrimental effects on egg traits.

## Background

Chicken egg providing dietary essentials have been used as a food by human beings since antiquity. The commercial table eggs contain a high proportion of ω-6 polyunsaturated fatty acids and dietary cholesterol but are poor source of ω-3 fatty acids. The lipids composition (30–40 %) of chicken egg has been an area of primary consumer concern, due to the relationship between specific dietary lipids and the development of coronary heart diseases, atherosclerosis, increasing stroke rate, high proportion of gallstones, enhancing depression rate and as a consequence is deleterious to human health and life expectancy [1, 2]. In the past decade, science has provided new information on the importance of the balance between ω-3 and ω-6 fatty acids in health and disease. The ability to enrich the eggs with polyunsaturated fatty acids has explored new awareness for a healthful food supply. Long chain ω-3 fatty acids; which are usually in short supply from the egg; can be manipulated by dietary means [3].

The full-fat flaxseed despite providing a source of protein (20–24 g/100 g), dietary fiber (25–28 g/100 g) and flaxseed oil (35–45 g/100 g) to laying hens has also obtained relatively new flourished concept as staple poultry feedstuff because of its high alpha-linolenic acid concentration. In recent years, many studies have looked at finding the best formulation of flaxseed supplemented feed that effectively raises the yolk’s n-3 fatty acid content in egg [4]. However, unprocessed flaxseed also encloses well documented anti-nutritional factors such as cyanogenic glycosides and tannin which are associated with growth depression, feed efficiency, egg production, egg weight, egg quality, abnormal respiration and nervousness of laying hens [5]. This situation demands more attention to reduce the toxic effect and improve the nutritional quality of flaxseed through effective and economical thermal processing. The detoxification of flaxseed meal can be achieved by boiling in water, microwave roasting, wet autoclaving or acid treatment [6]. The extrusion processing has been proposed as an effective approach to remove the anti-nutrients in raw materials. The extrusion cooking is highly adaptable and focused form of processing in which food or feedstuff is enforced to flow under controlled conditions of heating and shearing through a terminal die. The principal features of extrusion cooking take account of flexible product characteristics, high energy efficiency, less space required for operation, new feed products, automated control system and product quality with no effluent showing environmental-friendly [7, 8].

The sensory attributes such as smell, color, flavor, texture and overall acceptability are key determinants of food product development process. The consumer and sensory testing could help to answer questions related to characteristics, variation and acceptability of the product [9]. The animal food products such as eggs are very resistant to oxidative reactions due to presence of natural antioxidants, including, vitamin E, avidin, and phosphatine. However, the long chain ω-3 fatty acids possess a characteristic “fishy” or “paint-like” aroma, and so there is concern that alteration of ω-3 fatty acid content may affect the eating (sensory) quality of the food products [4, 10]. The objective of this research was to determine the effect of extruded flaxseed supplemented diets on hen performance, fatty acids composition and sensory attributes of resultant Bio-omega-3 eggs.

## Methods

### Hot extrusion processing of flaxseed

The flaxseed cv. *Chandni* was procured from Oilseeds Research Institute, Faisalabad, Pakistan. The seeds of the variety were cleaned to remove any debris or field dirt and any other extraneous matters. The alkaline titration method was used for the determination of cyanogenic contents in flaxseed samples [11]. The concentration of tannin in the flaxseed meal samples was measured by Folin–Denis method with minor modifications [12]. Commercially available single-screw extruder, Extru-tech E325 (Extru-tech, Sabetha, Kansas, USA), consisted of screws (83 mm diameter), barrel (254 mm diameter) and barrel length to diameter ratio of 9:1 was used for the detoxification of flaxseed. The extruder was operated at barrel exit temperature (140 °C), screw speed (160 rpm) and feed rate (25 kg/h). The extruded samples were cooled down to room temperature and placed in sealed polyethylene bags for further analysis.

### Experimental laying hens and diets

One hundred and sixty, Babcock laying hens (age 24 weeks old) were selected at random from a large flock at Department of Poultry Science, University of Agriculture, Faisalabad, Pakistan. The animals were treated by following guidelines of the ethical committee as approved by the university. Ten Babcock laying hens were placed in each of 16 wire-mesh pens and hens were exposed to 14 h light and 10 h dark day. The experimental diets prepared for laying hens contained major ingredients such as corn, wheat, rice polishing, canola meal, gluten 60 %, gluten 30 %, wheat bran, soybean meal, vegetable oil, dicalcium phosphate, limestone (ground) and vitamins/minerals premix. The extruded flaxseed meal was supplemented with different concentrations as 10 % (T_1_), 20 % (T_2_) and 30 % (T_3_) in poultry feed for laying hens. The diet without extruded flaxseed meal was kept as control (T_0_). The percentage composition of experimental diets has been given in Table 1. The diets were formulated to meet the daily nutrient requirements of the laying hens according to recommendations given by NRC [13] and to be equal in protein and metabolizable energy contents. The Babcock laying hens were randomly assigned to experimental diets (4 replicate pens for each diet). Feed and water were provided for *ad libitum* consumption through the eight weeks experimental period. The experimental area was maintained at temperature of 25 ± 2 °C with relative humidity 65 ± 5 %. The experimental hens were also subjected to routine management such as vaccination and medication.

**Table 1:** Experimental diets with percentage composition for Babcock laying hens

Ingredients	Experimental diet			
	Control	10 % EFM	20 % EFM	30 % EFM
Corn	35	35	35	20
Wheat	05	09	15	20
Rice polishing	15	10	05	-
Canola meal	15	10	05	-
EFM	-	10	20	30
Gluten 60 %	05	4.5	03	-
Gluten 30 %	-	-	-	5.5
Wheat bran	-	-	-	07
Soybean meal	08	08	08	08
Vegetable oil	08	04	-	-
Dicalcium phosphate	1.5	1.5	1.5	1.5
Lime stone (ground)	07	7.5	07	7.5
Vitamin/mineral premix	0.5	0.5	0.5	0.5
Analyzed nutrient content				
Crude protein (%)	16.9 ± 0.1a	17 ± 0.1a	17 ± 0.1a	17 ± 0.1a
Metabolizable energy (kcal/kg)	2838 ± 4a	2840 ± 3a	2844 ± 2a	2846 ± 3a
Crude fat (%)	10.1 ± 0.3a	10.2 ± 0.3a	10.2 ± 0.2a	10.3 ± 0.2a
^1^C_16:0_ *	12.1 ± 0.1a	9.6 ± 0.1b	7.6 ± 0.1c	6.3 ± 0.1d
^2^C_18:0_ *	16 ± 0.3a	13.2 ± 0.2b	9.4 ± 0.3c	7.4 ± 0.4d
^3^C_18:1_ *	28.6 ± 0.5a	23.5 ± 0.4c	23 ± 0.5c	17.6 ± 0.5d
^4^C_18:2_ *	38.2 ± 0.7d	38.9 ± 0.7c	41.5 ± 0.8b	42.8 ± 0.9a
^5^C_18:3_ *	2.7 ± 0.1e	8.2 ± 0.4d	14.2 ± 0.6c	21.1 ± 0.7b
^6^C_20:4_ *	0.6 ± 0.1a	0.4 ± 0.1a	0.3 ± 0.1a	0.2 ± 0.1a
^7^C_20:5_ *	1.5 ± 0.13a	1.3 ± 0.1a	1.1 ± 0.1a	1 ± 0.1a
^8^C_22:6_ *	0.5 ± 0.1a	0.3 ± 0.1a	0.3 ± 0.1a	0.2 ± 0.1a
Calculated nutrient content				
Calcium (%)	3.04a	3.12a	3.16a	3.25a
Available p (%)	0.59a	0.50a	0.55a	0.62a
Methonine (%)	0.30a	0.33a	0.35a	0.36a
Lysine (%)	0.78a	0.84a	0.82a	0.85a

Means sharing similar letters in a column for individual parameter do not differ significantly from one another (*p* > 0.05)

^1^Palmitic ^2^Stearic ^3^Oleic ^4^Linoleic ^5^Linolenic ^6^Arachadonic ^7^Eicosapentaenoic ^8^Docosahexaenoic

*EFM* Extruded flaxseed meal

*Percent of total fatty acids composition

### Chemical composition of experimental diets

The crude protein in each diet sample was determined by using Kjeltech Apparatus (Model: D-40599, Behr Labor Technik, Gmbh-Germany) as described in AACC [14] Method No. 46–10. The estimation of gross energy of experimental diets was carried out through Oxygen Bomb Calorimeter (IKA^®^-WERKE C 2000 Basic Version) consisting of decomposition vessel and cooling water handling system (KV 600 Thermostat) following the procedure described by Harris [15]. The crude fat content in diet samples was determined by using soxhlet apparatus (Model: H-2 1045 Extraction Unit, Hoganas, Sweden) according to AACC [14] Method No. 30–25. The fatty acids profile of extracted oil from diet samples was carried out according to the method described in AOCS [16] Method No. Ce 1f-96.

### Performance of hens and collection of egg samples

The laying hens were subjected to performance characteristics for feed consumption, body weight, egg production and egg weight during 24–32 weeks of hen age. The feed consumption was recorded by calculating total feed provided to the bird deducting the total feed remained uneaten at the end of each week. The body weight of experimental hens was recorded at the initiation of experiment followed by the weight recorded at the end of each week to keep record of change in body weight till the expiration of the experiment. The eggs were collected twice a day at 10:00 am and at 2:00 pm to calculate egg production daily for each replicate. The eggs laid by hens subjected to each treatment were weighed daily and average mean value of eggs weight was calculated weekly.

### Chemical analysis of eggs

The total lipids of experimental eggs were determined gravimetrically according to the AOAC [17] Method No. 923.07. The quantitative estimation of cholesterol content in the eggs was carried out by the method as described by Fenton and Sim [18].

The extraction of lipids from egg yolk sample was analyzed according to the method of Folch et al. [19] for the determination of fatty acids composition. The esters of fatty acids in each sample were prepared and analyzed through Gas Chromatograph (Varian 3900) apparatus equipped with an auto sampler, flame-ionization detector (FID) and supelco wax column (30 m x 0.25 μm film coating) according to AOCS [16] Method No. Ce 1f-96 as described previously.

### Organoleptic evaluation of eggs

The sensory evaluation of hard cooked eggs was carried out in an adequate room (25 °C) according to the instructions given by Gonzalez-Esquerra and Leeson [20] on a 10-point hedonic scale. 15 eggs randomly selected per treatment were stored at 5 °C for 14 days, boiled for 15 min, kept in warm water (35 °C) until served to fourteen panel judges consisting of experienced and untrained panelists. Each judge gave written informed consent after explanation of risks and benefits of participation prior to the study. Each panelist was offered with unsalted crackers, water and hard boiled peeled eggs randomly from experimental treatments placed in closed plastic dishes labeled with three secret digit codes. The panelists were provided informative instructions and brief definitions of attributes such as aroma, taste, aftertaste, flavor, presence of off flavors and overall acceptability. Each panelist was asked to list their preference on a 10-cm line (1 = dislike extremely to 10 = like extremely) twice a week for 2 consecutive weeks.

### Statistical analysis

The data obtained for each parameter was subjected to statistical analysis to determine the level of significance by using the software package (Statistic 8.1) according to the method described [21]. The Duncan’s multiple range (DMR) test was used to estimate the level of significance that existed between the mean values.

## Results

The raw flaxseed possessed HCN (198 ± 0.6 mg/kg) and tannin (146 ± 0.5 mg/100 g), respectively. After heat treatment, the extruded flaxseed meal contained 86 % and 76 % less HCN and tannin compounds than the initial material. The protein, lipid and metabolizable energy contents in experimental diets ranged between 16.9 ± 0.1 %–17 ± 0.1 %, 10.1 ± 0.3 %–10.3 ± 0.2 % and 2838 ± 4 kcal/kg–2846 ± 3 kcal/kg, respectively (Table 1). The composition of saturated and unsaturated fatty acids varied significantly among different experimental diets. The fatty acids found in control diet were more saturated as compared to found in extruded flaxseed meal manipulated diets. The palmitic, stearic, oleic_,_ linoleic and alpha-linolenic acid contents in control diet were found 12.1 ± 0.1 %, 16 ± 0.3 %, 28.6 ± 0.5 %, 38.2 ± 0.7 % and 2.7 ± 0.1 %, respectively. The palmitic, stearic, oleic_,_ linoleic and alpha-linolenic acid contents in extruded flaxseed experimental diets varied from 6.3 ± 0.1 %–9.6 ± 0.1 %, 7.4 ± 0.4 %–13.2 ± 0.2 %, 17.6 ± 0.5 %–23.5 ± 0.4 %, 38.9 ± 0.7 %–42.8 ± 0.9 % and 8.2 ± 0.4 %–21.1 ± 0.7 %, respectively. The long chain polyunsaturated fatty acids (arachadonic, eicosapentaenoic and docosahexaenoic acid) did not differ significantly among different experimental diets (Table 1).

The feed consumption, body weight and egg production rate of laying hens fed on control diet differed significantly from the laying hens fed on various levels of extruded flaxseed supplemented diets (Table 2). The data also substianted the declining trend in these parameters with increasing levels of supplemented extruded flaxseed. The results further revealed that laying hens fed on 30 % extruded flaxseed supplemented diets produced significantly heavy weight eggs than from the laying hens fed on 10 % and 20 % levels of extruded flaxseed supplemented diets (Table 3).

**Table 2:** Effect of experimental diets on hen performance and biochemical constituents of eggs

Performance parameter	Experimental diet			
	Control	EFM (10 %)	EFM (20 %)	EFM (30 %)
Feed consumption (g/bird/day)	108 ± 2a	104 ± 2b	102 ± 1c	101 ± 1d
Body weight (g)	1613 ± 7a	1607 ± 8a	1593 ± 9b	1546 ± 10c
Egg production (%)	93 ± 3a	85 ± 3b	78 ± 2c	64 ± 4d
Egg weight (g)	61.5 ± 0.9a	61.6 ± 1.0a	62 ± 1.1ab	62.3 ± 1.1b
Biochemical constituent				
Total lipids (g/100 g)	27.9 ± 0.1a	27.9 ± 0.1a	27.9 ± 0.1a	27.8 ± 0.1a
Cholesterol (mg/egg)	218 ± 2a	216 ± 3a	214 ± 3a	214 ± 3a
Oleic acid (%)*	36 ± 0.1a	34.7 ± 0.2b	32.1 ± 0.1c	30 ± 0.1d
Linoleic acid (%)*	11.1 ± 0.1d	12.4 ± 0.1c	13.4 ± 0.1b	14.2 ± 0.1a
Linolenic acid (%)*	1 ± 0.1d	6.3 ± 0.3c	9.6 ± 0.4b	13.5 ± 0.3a
Arachidonic acid (%)*	1.7 ± 0.1a	1 ± 0.2b	0.7 ± 0.1c	0.5 ± 0.1d
Docosahexaenoic acid (%)*	0.1 ± 0.1c	0.5 ± 0.1b	0.8 ± 0.1a	0.9 ± 0.1a
Σω-6 to Σω-3 fatty acids ratio	9.6 ± 0.2a	4.1 ± 0.1b	3.4 ± 0.2c	2.3 ± 0.1d

*EFM* Extruded flaxseed meal

Means sharing similar letters in a row for individual parameter do not differ significantly from one another (*p* > 0.05)

*Percent of total fatty acids composition

**Table 3:** Comparison of experimental diets (significance) for hen productivity parameters and fatty acids profile of eggs

Experimental Diet (Comparison Pair)	Hen productivity parameters				Fatty acids profile of eggs					
	FC	BW	EP	EW	Oleic acid	Linoleic acid	Linolenic acid	Arachidonic acid	Docosahexaenoic acid	Σω-6 to Σω-3 fatty acids ratio
Control vs. EFM (10 %)	NS	NS	NS	NS	*	NS	*	*	*	**
Control vs. EFM (20 %)	*	NS	NS	*	**	*	**	*	**	**
Control vs. EFM (30 %)	*	***	**	*	**	**	***	*	**	**
EFM (10 %) vs. EFM (20 %)	NS	NS	NS	NS	*	NS	NS	NS	*	NS
EFM (10 %) vs. EFM (30 %)	NS	***	*	*	**	*	*	*	*	*
EFM (20 %) vs. EFM (30 %)	NS	NS	NS	NS	*	NS	NS	NS	NS	NS

* = Significant (p ≤ 0.05) ** = Significant (p ≤ 0.01) *** = Significant (p ≤ 0.001) NS = Non-significant (*p* > 0.05)

*EFM* Extruded flaxseed meal

*FC* Feed consumption (g/bird/day)

*EP* Egg production (%)

*EW* Egg weight (g)

*BW* Body weight (g)

The total lipids in egg yolk of laying hens fed on experimental diets varied from 27.8 ± 0.1 g/100 g to 27.9 ± 0.1 g/100 g. The cholesterol content in different experimental egg yolks did not vary significantly due to differences in experimental diets (Table 2). The laying hens fed on control diet possessed higher deposition of oleic acid in egg yolk than laying hens fed on extruded flaxseed supplemented diets. The laying hens fed on 30 % extruded flaxseed supplemented diets showed maximum deposition of linoleic acid i.e., 14.2 ± 0.1 %. The α-linolenic acid deposition in egg yolk of laying hens increased significantly with an increase in extruded flaxseed level. The laying hens fed on extruded flaxseed supplemented diets showed more than 8-fold deposition of α-linolenic acid in egg yolk as compared to laying hens fed on control diet. Furthermore, this value doubled when laying hens were fed on 30 % extruded flaxseed supplemented diets. A declining trend in arachidonic acid deposition in egg yolk of laying hens fed on extruded flaxseed supplemented diets was observed. There was an increasing trend in deposition of docosahexaenoic acid in egg yolk with increase in supplementation of extruded flaxseed in experimental diet. The laying hens fed on control experimental diet showed significantly higher ratio of ω-6 to ω-3 fatty acids in egg yolk of laying hens. There was also a decreasing trend in ratio of ω-6 to ω-3 fatty acids in egg yolk of laying hens with an increase in supplementation of extruded flaxseed in experimental diet (Table 2).

**Fig. 1:**
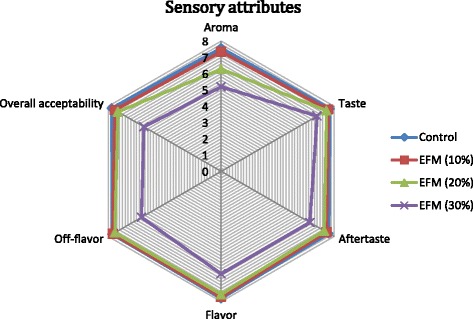
Comparison of experimental diets for organoleptic acceptability of resultant Bio-omega-3 eggs

The scores assigned to sensory attributes of eggs were significantly affected due to compositional variation in diets (Fig. 1). The sensory scores given to eggs obtained from laying hens fed on control diet were significantly higher than those obtained from the laying hens fed on various levels of extruded flaxseed supplemented diets. The majority of untrained panelists were unable to determine any differences in sensory attributes between control and ω-3 enriched eggs produced by hens fed with 10 % extruded flaxseed supplemented diet. The sensory scores of eggs decreased with an increase of extruded flaxseed level in diet. The lowest sensory scores were given to experimental eggs obtained from laying hens fed on 30 % extruded flaxseed supplemented diets.

## Discussion

### Detoxification of flaxseed meal

The characterization of raw material helps to determine the suitability for preparation of feed or other products. The concentration of HCN has been the subject of extensive investigation as contributed by Yang et al. [22] who found HCN 157.6 mg/kg in flaxseed meal. Wanasundara and Shahidi [23] reported condensed tannin in the range of 130 to 136 mg/100 g flaxseed meal. The optimized results for barrel temperature of 146.0 °C and feed rate of 32.7 kg/h exhibited maximum rate of HCN removal (93.2 %) during the twin-screw extrusion detoxification technique on flaxseed via stepwise non-linear response surface methodology [24]. Mukhopadhyay et al. [25] has also predicted maximum reduction 61.2 % of tannin in linseed meal at optimum values of barrel exit temperature 82.5 °C and screw speed 90 rpm.

### Nutrient composition of diets

The dietary ingredients either exceeding or declining from standards can affect metabolism and regular physiological processes of animal body. The nutrient content assessment is of great importance for estimation of digestibility and bioavailability of feed constituents. The undefined nutrient contents in self-mixed rations negatively affect the productivity performance and quality of laying eggs. The nutrient contents found in different experimental diets are in line with Jia et al. [5] and Hayat et al. [26]. The wide variations in saturated fatty acids contents may be due to difference in fatty acids composition of ingredients (extruded flaxseed) present in experimental diets. The study of Basmacioglu et al. [27]; Sosin et al. [28] and Sujatha and Narahari [29] showed similar contents of fatty acids in diets prepared for laying hens. The level of flaxseed, flaxseed composition and dietary ingredients used in feed formulations may be linked with wide variations in fatty acids composition of experimental diets prepared for laying hens to produce long chain ω-3 fatty acids-enriched eggs.

### Performance of Babcock laying hens

A decrease in consumption of flaxseed supplemented experimental diets, hen body weight and resultant egg production has also been reported in different study trials [5, 26]. Egg production has been characterized as main attribute that reveal growth and maturity index of laying hens. Egg production may be affected by such factors as feed consumption and body weight of laying hens [30, 31]. The feed consumed by the laying hens is used mainly for body maintenance. Nutrients in excess of those necessary for maintenance can be used for egg production. The difference in egg production among experimental laying hens fed on extruded flaxseed supplemented diet may be due to variation in amount of feed consumption. Egg weight is one of very important criteria to evaluate the performance of laying hens. Nutritional characteristics of the diet define a major role to change the position and requirement of laying hens to produce eggs with unique egg weight profile. The Babcock laying hens fed with control diet maintained their normal egg weight production according to their body weight and sexual maturity period. The laying hens with low body weight tend to produce fewer eggs. The nutrient composition and substantial amount of phytochemicals and phytoestrogens in diet also reflect the egg production rate [32]. The presence of some anti-nutritional compounds such as tannin and cyanogenic compounds may decrease production of endogenous enzymes released from pancreas and reduce the productivity of laying hens [33]. The egg weight depends largely upon the level of flaxseed supplementation in diet [26, 34]. Leeson et al. [35] reported production of heavy weight eggs from laying hens fed on flaxseed enriched diets. The change in availability of nutritional profile may significantly alter the egg weight. The differences in egg weight of laying hens fed on different experimental diets may be due to variations in the availability of protein contents which has been identified as most limiting factor for egg weight [36]. The extrusion cooking of flaxseed increases the protein-amino acid bioavailability by reduction of anti-nutritional compounds which may result in production of heavy weight eggs. The maximum derived energy from experimental diet also develop an abnormal ovarian structure and ultimately depress the egg production rate and increases the egg weight of laying hens [37].

### Biochemical analysis of egg yolk

Egg yolk provides lipids, fatty acids and proteins required for embryonic growth. Egg analytical measurements generally involve determination of total lipids, fatty acids composition and cholesterol content of yolk. The lipids recognized as biochemical and organic group contain a wide range of compounds that are structurally different from each other. Cholesterol is a member of family generically known as steroids [38]. Non-significant results with respect to total lipids found in egg yolks of experimental laying hens fed on different extruded flaxseed supplemented diets are supported by Cherian [39] and Hayat et al. [26]. The changes in total lipids of egg yolk through diet manipulation have been reported to be limited [40] which are also in partial agreement to results found in the present study. The cholesterol content of eggs have received more attention than before from consumers, medical professionals and researchers. Higher cholesterol content in eggs is of primary concern due to evidence of epidemiological studies to elevated plasma cholesterol levels and coronary artery disease. Non-significant results regarding cholesterol content in egg yolk of experimental hens fed to different extruded flaxseed supplemented diets are similar to the studies of Beynen [41] and Yalçýn et al. [42] . The genetic influence is also an important factor for modification of cholesterol contents in egg yolk [43]. Many researchers studied the effects of experimental diets rich in saturated and unsaturated fatty acids on cholesterol content in eggs, however they concluded that the reduction of cholesterol content through diet manipulation was found to be very limited [4].

The fatty acids composition of food products must be investigated due to their importance for human metabolism. The results found in the present study are consistent with the research findings of Scheideler et al. [44] who reported higher deposition of oleic acid in egg yolk of laying hens fed on control diet as compared to laying hens fed on flaxseed supplemented diet. The polyunsaturated fatty acids inhibit the activity of Δ^9^-desaturase enzyme, which may be involved in monounsaturated fatty acids synthesis [45, 46]. Caston and Leeson [47] reported higher deposition of linoleic acid in egg yolk of laying hens fed on raw flaxseed supplemented diet as compared to laying hens fed on control diet. The laying hens fed on flaxseed enriched diets produced egg yolk enriched with α-linolenic acid [4, 41]. Bean and Leeson [48] reported significantly increased deposition of α-linolenic acid in egg yolk from laying hens fed on extruded flaxseed (extruded at 149 °C for 15 to 20 s) which confirms the results found in the present study. The concomitant decrease deposition rate of arachidonic acid in egg yolk of laying hens was consistent with research findings by Caston and Leeson [47]; Cherian and Sim [49] and Aymond and Van Elswyk [50]. The enzymatic pathway for the synthesis of arachidonic acid from oleic acid is shared by the ω-3 fatty acids. The higher contents of longer chain ω-3 fatty acids, such as α-linolenic acid, eicosapentaenoic and docosahexaenoic acid inhibits the ∆^6^ desaturase and thereby reduces conversion of oleic to arachidonic acid [5, 51]. The poor conversion rate of α-linolenic to docosahexaenoic acid probably relates to the complexity of docosahexaenoic acid biosynthesis. The biosynthesis of polyunsaturated fatty acids is more complex because of enzymes availability from more than one intracellular compartment which is required for synthesis of 22-carbon polyunsaturated fatty acid with their first double bond at position 4 in a partial degradation-resynthesis cycle [47, 50, 52]. The laying hens fed on flaxseed enriched diet possessed lower ratio of Σω-6 to Σω-3 fatty acids in egg yolk of laying hens [53, 54].

### Sensory attributes evaluation

The supplementation of flaxseed meal in diet of laying hens decreased the sensory scores of hard-boiled eggs [55, 56]. Ferrier and co-workers [57] reported a decrease in acceptability scores regarding aftertaste of experimental eggs with an increase of flaxseed supplementation in diets for laying hens. Scheideler et al. [58] reported higher scores for flavor of experimental eggs obtained from laying hens fed on control diet than those obtained from laying hens fed on 15 % flaxseed supplemented diet. No adverse fishy flavor was noticed in hard cooked eggs produced from laying hens fed on 9 % flaxseed supplemented diet [59]. The panelists referred to eggs from hens fed on 30 % extruded flaxseed supplemented diet as being “fishy”. Twenty-eight male volunteers claimed a fishy or fish-product related flavor in eggs from hens fed on diet containing 20 % flaxseed [57]. The lipid oxidation produces off-flavor or “fishy” flavor and lead to reduction in acceptance of experimental eggs obtained from laying hens fed on diets supplemented with higher levels of flaxseed [60]. Many researchers have reported a decrease in overall acceptability of hard boiled eggs obtained from laying hens fed on flaxseed supplemented diet than those obtained from laying hens fed on control diet [61]. However, difference in overall acceptability of eggs obtained from laying hens fed on either 10 % or 20 % flaxseed was found to be non-significant which is in well agreement to the results found in the present study. The literature regarding sensory attributes and overall acceptability of ω-3 fatty acids-enriched eggs demonstrate that the feeding of flaxseed as a potential alternative feed ingredient can be characterized with greater stability and a slower onset of rancidity for resultant eggs. The dietary factors such as flaxseed variety, level of inclusion, type of antioxidant and egg sample preparation method may be responsible for reported differences in the sensory quality of ω-3 fatty acids-enriched eggs.

## Conclusions

The results of the present study conclude that extruded flaxseed can be targeted as a chief source of α-linolenic acid for supplementation in hen diet and production of designer eggs. The resultant modified ω-3 fatty acids-enriched egg can be a convenient way to supply enhanced level of essential fatty acids in the diet for the discerning consumers.
